# Draft Genome Sequence of a Pathogenic *Vibrio vulnificus* Strain Isolated in Brazil

**DOI:** 10.1128/genomeA.01274-17

**Published:** 2018-02-15

**Authors:** Monique Ribeiro Tiba-Casas, Eneida Gonçalves Lemes-Marques, Elisabete Aparecida Almeida, Flávia Barrosa Soares, Carlos Henrique Camargo

**Affiliations:** a Bacteriology Division, Instituto Adolfo Lutz, São Paulo, Brazil; b Campinas Center of Regional Laboratories, Instituto Adolfo Lutz, São Paulo, Brazil

## Abstract

We describe the draft genome sequence of the clinical *Vibrio vulnificus* strain 03_7315, isolated in 2016 from the blood of a diabetic patient who died of septicemia after ingestion of seafood. The draft genome, with 4,755,588 bp covering two chromosomes, presented 4,434 genes, 4,213 coding sequences, and 117 pseudogenes.

## GENOME ANNOUNCEMENT

*Vibrio vulnificus* is an opportunistic human pathogen responsible for most seafood-associated deaths worldwide. It is an important etiologic agent which may cause severe wound infections that can be fatal, require amputation, or lead to sepsis in susceptible individuals (1–3). *V. vulnificus* is a highly diverse species classified into three biotypes. Biotype 1 is responsible for most human infections, biotype 2 primarily affects fish, and biotype 3, although rarely reported, is responsible for human infections and may cause serious infections requiring amputation (3–6).

Here we describe the draft genome sequence of the clinical *V. vulnificus* strain 03_7315, isolated in 2016 from blood from a patient with diabetes mellitus, who died of septicemia after ingestion of seafood. Initially, identification of the bacterium was carried out by conventional biochemical methods, as well as PCR. The strain was subjected to whole-genome shotgun sequencing.

Genomic DNA was extracted according to the manufacturer’s instructions using the Qiagen DNeasy genomic DNA prep kit for Gram-negative bacterial cultures. DNA libraries were generated by the Strategic Laboratory, Adolfo Lutz Institute, and sequenced using the Life Technologies Ion Torrent S5. The 520 Chip paired with a 200-bp library was chosen for maximum coverage. SPAdes v.3.1 was used to assemble each of the genomes. Ion Torrent sequencing generated 3,146,105 reads with coverage of 165×. Reads were *de novo* assembled to generate 115 contigs of ≥500 bp, with an *N*_50_ value of 135,243 bases and the longest contig size of 347,067 bases. The draft genome of *Vibrio vulnificus* 03_7315, with 4,755,588 bp, covering two chromosomes and containing 4,434 genes, 4,213 coding sequences, 117 pseudogenes, was predicted and annotated by the NCBI Prokaryotic Genome Annotation Pipeline.

There is a paucity of epidemiological and genotypic data on *V. vulnificus* from Brazil, a continental country with a warm coast longer than 7,300 km. The shotgun-sequenced 03_7315 strain provides novel information for future studies on the epidemiology of *Vibrio vulnificus* circulating in Brazil, a very pathogenic bacterium with case fatality rates exceeding 50%.

### Accession number(s).

The whole-genome shotgun projects reported here have been deposited at DDBJ/EMBL/GenBank under the accession number NWBS00000000.
