# Correction for Tiba-Casas et al., “Draft Genome Sequence of a Pathogenic Vibrio vulnificus Strain Isolated in Brazil”

**DOI:** 10.1128/genomeA.00243-18

**Published:** 2018-03-29

**Authors:** Monique Ribeiro Tiba-Casas, Eneida Gonçalves Lemes-Marques, Elisabete Aparecida Almeida, Flávia Barrosa Soares, Carlos Henrique Camargo

**Affiliations:** a Bacteriology Division, Instituto Adolfo Lutz, São Paulo, Brazil; b Campinas Center of Regional Laboratories, Instituto Adolfo Lutz, São Paulo, Brazil

## AUTHOR CORRECTION

Volume 6, no. 7, e01274-17, 2018, https://doi.org/10.1128/genomeA.01274-17. Page 1: The following statement should be added to Acknowledgments. “Publication of this study was supported by grant no. 2017/22402-4, São Paulo Research Foundation (FAPESP).”

